# Mechanisms of alignment of lamellar-forming block copolymer under shear flow†

**DOI:** 10.1039/d4sm01241k

**Published:** 2024-12-16

**Authors:** Marco Pinna, Javier Diaz, Christopher Denison, Andrei Zvelindovsky, Ignacio Pagonabarraga

**Affiliations:** a School of Mathematics and Physics, College of Health and Science, Centre for Computational Physics, University of Lincoln, Brayford Pool Lincoln LN6 7TS UK mpinna@lincoln.ac.uk; b Departament de Física de la Matèria Condensada, Universitat de Barcelona, Martí i Franqués 1 08028 Barcelona Spain jdiazbranas@ub.edu; c Universitat de Barcelona Institute of Complex Systems (UBICS), Universitat de Barcelona 08028 Barcelona Spain

## Abstract

The potential applications of block copolymer thin films, utilising their self-assembly capabilities, are enhanced when achieving long-range ordering. In this study we explain the experimental alignment of lamellae under shear flow findings [S. Pujari *et al. Soft Matter*, 2012, **8**, 5258] and classify the alignment mechanisms based on shear rate and segregation, uncovering similarities to the systems subjected to electric fields, suggesting a common pathway of lamellae orientations. However, the presence of thin films surfaces introduces distinct features in the lamellae orientation under shear compared to electric fields. Notably, we observe the emergence of a three-dimensional rotation alongside the conventional two-dimensional rotation. Furthermore, a transient regime has been identified within the melting mechanism, which confirms the existence of the checkboard pattern proposed by Schneider *et al.* [*Macromolecules*, 2018, **51**, 4642]. These findings significantly enhance our understanding of block copolymer alignments and shed light on the intricate interplay between external fields and the lamellar structure.

## Abstract 2

The toc picture shows 3D lamellae reorienting from the starting configuration (left) to a mix of parallel and perpendicular lamellae (to the shear flow plane) after shear flow rate (right). Shear flow velocity is along the *x*-direction, and the velocity gradient is along the *z*-direction.

## Graphical abstract

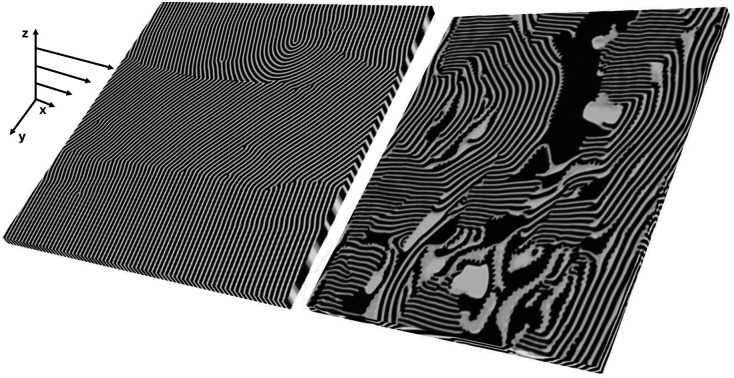

## 1 Introduction

**Fig. 1:**
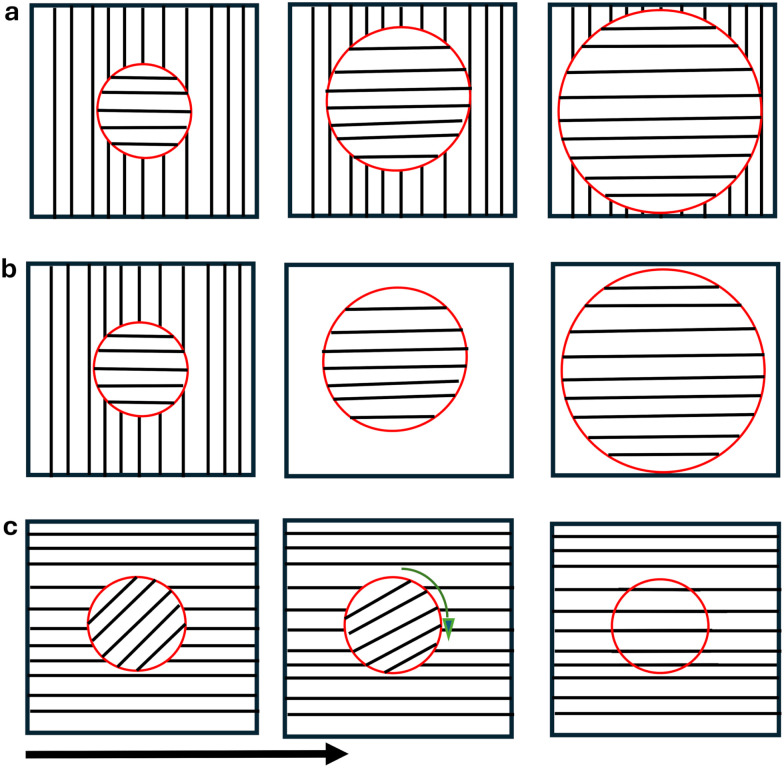
Different mechanisms of alignment of a lamellar system: (a) nucleation and growth of stable domains or shrinking of the unstable ones; (b) partial melting of the unstable domains; (c) rotation of the unstable domains. The arrow indicates the direction of the applied external field.

Block copolymers (BCP) are chain molecules which are composed of chemically different blocks covalently connected in one macromolecule. Thanks to their ability to self-assemble into different structures on a scale of 10–100 nm, they have been used in recent years for the miniaturisation of devices and electronic components,^1–5^ for production of masks for nanolithography, and fabrication of nanoporous membranes for advanced separation media and photonic crystals.^1,6–12^ In bulk, they can self-assemble into different morphologies such as lamellae, hexagonally packed cylinders, body-centred-cubic spheres and more complex morphologies such as gyroid. Most experimental and theoretical studies focus their attention on BCP confined in thin films. In bulk the morphology of BCPs is mainly determined by the molecular architecture and by the interaction between the different components. In thin films, instead, the surface interaction plays an important role.^1,13^ In the simplest case of a symmetric lamellar di-BCP, if a surface exhibits an energetic preference for one of the two blocks, then the lamellae will orient parallel to the surface. Instead, if the surface has no preferential blocks, the lamellae will be perpendicular to the surface. The formation of lamellae perpendicular to the surface of the film is desiderable for some lithography process and nanowire grids.^14^ Moreover, the thickness of the film plays a crucial role: if the thickness is incommensurate with the bulk lamellar period *H*_0_, the frustration of the system will change the lamellar conformation giving new morphologies. New structures have been found in thin film confinement, such as perforated lamellae,^15–17^ parallel and perpendicular lamellae, and cylinders parallel and perpendicular with respect to the wall.^18–20^ The global ordering of lamellar domains is often diminished by the presence of defects, grain boundaries and a lack of long range control over the alignment of the domains. The control of long range ordering of di-BCPs is very important for practical applications in chemistry, material science and nanotechnology.^14^ Tailoring film structures is a very active area of current research. External fields such as an electric field, shear flow, chemical pattern and surface preferences have often been used to obtain long-range order.^20–27^ It has also been shown, using an external field such like an electric field, both experimentally and theoretically, that the alignment mechanism on lamellar morphology happens following three distinct mechanisms: rotation, nucleation and growth and a partial melting of the system (see Fig. 1).^28–30^ The mechanism of partial melting was found in the first instance computationally, with a simple Ginzburg–Landau description^31,32^ and only later confirmed by a more sophisticated method such as dynamic self-consistent field theory^33^ and afterwards confirmed by the experiments.^34^

Experimentally, long range orientation applying oscillatory shear on poly(ethylene-propylene)-poly(ethylethylene) (PEPPEE) diblock copolymer melts (in bulk systems) has been obtained by Koppi *et al.*^35,36^ They found two distinct orientations: lamellae oriented perpendicular to the shear plane and coincident with the shear direction, and lamellae parallel to the plane of shear. These orientations were dependent on the polymer chemistry, frequency and temperature of the studied system.

Later, register's group studied experimentally the lamellae morphology in neutral ultra-thin films under a shear flow. They found alignment of perpendicular lamellae to the shear plane along the direction of the applied shear flow.^37^ Computationally such orientation has been obtained in bulk by dynamic density functional theory.^38^ The stability of this configuration has been studied, in bulk, using dissipative particle dynamics (DPD).^39^ In this study, they found that for higher shear rates the long-range orientational order of the lamellae inside the grains disintegrates while at lower shear rates there is a shrinking of the unstable grains.^39^ Experimentally, it has also been demonstrated that a stress level exceeding 10 KPa is necessary to align the lamellae in the direction of shear flow, even when the distance between domains is less than one. In the case of thicker layers, researchers have noted the presence of a polystyrene layer at the top of the film (parallel lamellae), which can be eliminated by etching the film.^37^

In this work, we will perform a systematic study of the aligning mechanism of lamellar-forming BCP in ultra-thin neutral films under shear flow. The thickness of the film will be explored in order to understand the appearance of parallel lamellar orientation in experiments of thicker films,^37^ as well as the degree of segregation of the lamellar domains. A mesoscopic cell dynamic simulation (CDS) method will be used to capture the dynamic behaviour of BCP under shear stress. We use a simple model where the hydrodynamic interactions are neglected in this work. In a weakly segregated system, the interfaces are diffuse and the viscosity variation is minimal.^40^ Thus, the linear velocity profile is a first approximation, disregarding viscosity mismatches and interfacial tension-driven flows. The general solution for the velocity field can be obtained from hydrodynamics. It has been shown that for a layered (lamellae) system with different polymer viscosities, the solution adopts the linear velocity profile.^40^ This refinement is essential for describing the temperature-dependent orientation of the mesostructure lattice in the gradient-vorticity plane at high shear rates (see ref. 41). Nevertheless, in the weak segregation regime, the main aspects of flow alignment can be accurately described by a diffusion-convection equation with the imposed velocity profile from eqn (3). Numerous studies have demonstrated that this equation effectively captures many relevant phenomena in sheared inhomogeneous fluids.,^40–55^ Furthermore, if the polymers have equal bulk viscosity, the approximation is even more accurate. Using this approximation, we have a model that is considerably fast allowing us to explore large systems and phase diagrams where the migration of defects plays an important role to the different mechanisms. This particular theoretical model has an established record on reproducing experimental results as seen in ref. 15 and 55–57.

## 2 Model

The BCP melt is described by differences in concentration of A and B monomers, *ϕ*_A_ and *ϕ*_B_ respectively, leading to the local order parameter *ψ*(**r**,*t*) = *ϕ*_A_(**r**,*t*) − *ϕ*_B_(**r**,*t*) + (1 − 2*f*) where *f* = *N*_A_/(*N*_A_ + *N*_B_) is the overall fraction of A monomers in the system. In this work we restrict to a lamellar-forming symmetric BCP and set *f* = 0.5. The free energy of the system is given by the Ohta–Kawasaki functional^58^ for symmetric melts1
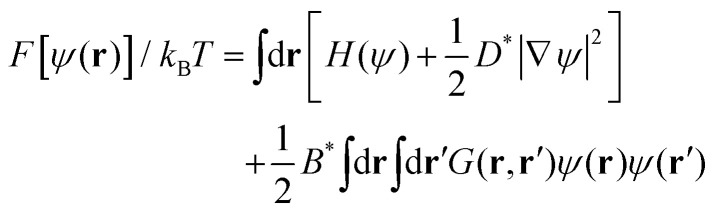
where the local free energy density is2
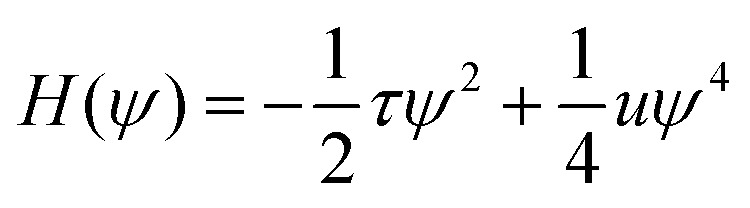
which determines the equilibrium values of the order parameter, *i.e.* the amplitude of the fluctuations, 
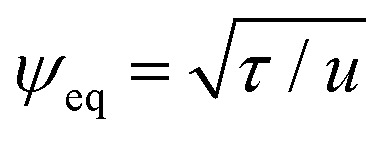
. Parameter *τ* ≈ (*χN* − 3.6)/2*N* is related to the Flory–Huggins *χ* parameter of the melt and the degree of polymerisation *N*, and therefore is inversely proportional to the temperature.^58^ Meanwhile, *u* is a phenomenological parameter. The square gradient term prefactor^58^D* = *b*^2^/12 is related to the Kuhn length *b* and controls the interface width 
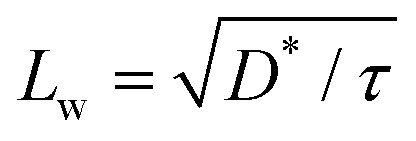
. The connectivity of the BCP chain is introduced in the long-range free energy term with the Green function of the Laplacian ∇^2^*G*(*r*) = −*δ*(*r*). The prefactor B* = 36/(*Nb*)^2^ controls the length of the BCP chain and therefore is related to the lamellar periodicity.^58^ Magnitudes with asterisk D* and B* have explicit dimensions in powers of length, and can be directly mapped to microscopic properties of the BCP chain.

**Fig. 2:**
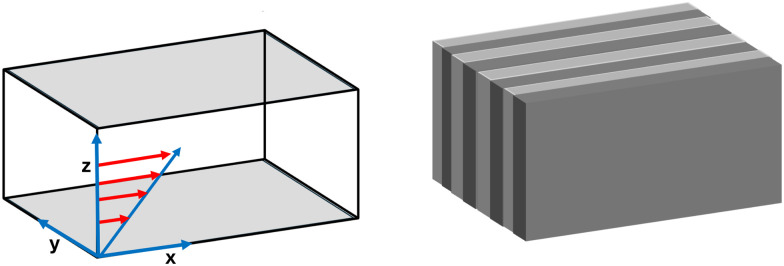
The figure on the left shows a simple 3D geometrical representation of the studied system. The grey plane (representing the shear plane) corresponds to the *x*–*y* plane, where the neutral walls of the thin film system are located. In this configuration, the gradient of the shear is oriented along the *z*-direction. The neutral plane lies in the *y*–*z* plane, while the vorticity plane is positioned in the *x*–*z* plane. The figure on the right illustrates the stable lamellae configuration formed after the applied shear.

A shear flow with velocity field **v** = (*v*_*x*_,*v*_*y*_,*v*_*z*_) is considered, leading to the modified Cahn–Hilliard dynamics^43,59^3
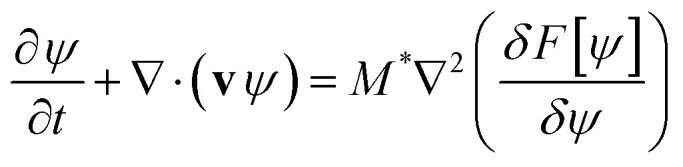
where M* is a mobility parameter quantifying the diffusivity of the melt, which can be related to microscopic properties of the chain.^60^ A steady shear flow with velocity components *v*_*x*_(*z*) = γ̇*z, *v*_*y*_ = *v*_*z*_ = 0 is considered (see Fig. 2-left side). The linear velocity profile in eqn (3) is an exact solution to the hydrodynamics problem only in a disordered (homogeneous) polymer system. Considering the complexity of the full hydrodynamic solution and the ability to achieve a weakly segregated state by adjusting temperature, this approximation is a suitable starting point for investigating systems comparable in size to those studied and used in experiments.

Eqn (3) can be made explicit as4
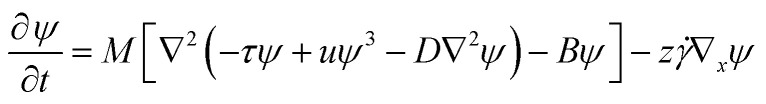
where ∇_*x*_ is the spatial derivative along the *x* Cartesian coordinate. Eqn (4) is written is simulation units with unit of length *L* and time *T*, leading to parameters *D* = D*L^−2^, *B* = B*L^2^, *M* = M*TL^−2^ and ** = ***T. They can be related to microscopic properties of the BCP chain 
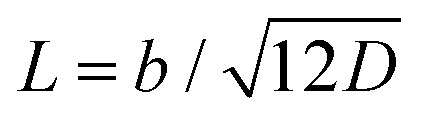
 and *T* = (*M*/M*)*L*^2^. A Weissemberg number can be extracted

5 Wi = *t*_c_*γ̇*

that compares the relaxation of the BCP chain^59^*t*_c_ = *L*_w_^2^/(*Mτ*), with the shear flow time scale *γ̇*^−1^.

Periodic boundary conditions are imposed across the *x* and the *y* horizontal directions, while hard walls are placed at the top and bottom of the system (*z* = 0 and *z* = *L*_*z*_). Dirichlet boundary conditions have been imposed on *ψ*, with *ψ*(0) = *ψ*^bottom^_s_ and *ψ*(*L*_*z*_) = *ψ*^top^_s_ and a Neumann boundary conditions for the chemical potential *μ*(**r**) = δ*F*/δ*ψ*(**r**) at the surface δ*μ*/δ*z* (0) = δ*μ*/δ*z*(*L*_*z*_) = 0. For simplicity we will limit to the case of *ψ*^bottom^_s_ = *ψ*^top^_s_ = *ψ*_s_ that has identical walls (see Fig. 2-left side). Fig. 2 on the left side shows the 3D studied system while the right side shows the obtained stable lamellae configuration once the steady shear flow has been applied.

**Fig. 3:**
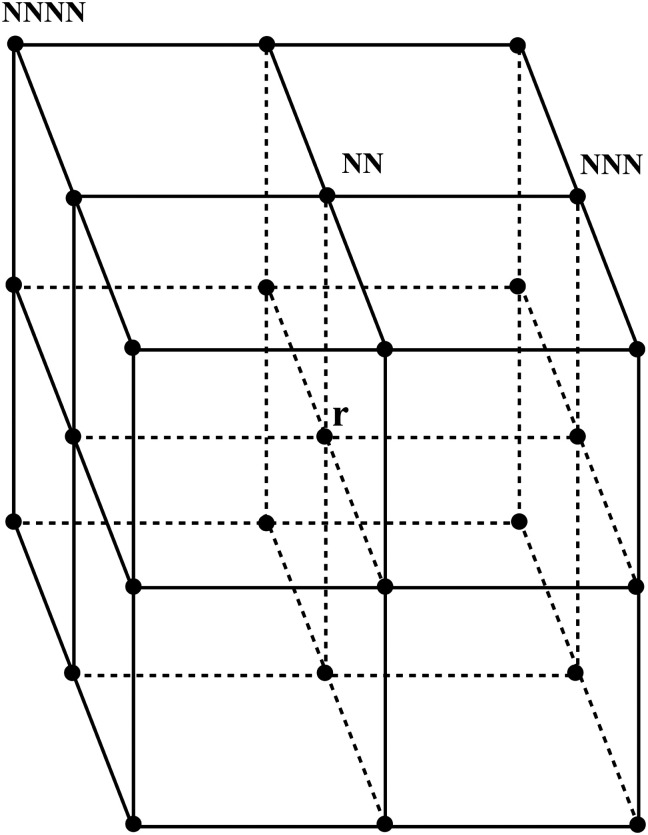
A stencil for Laplacian, where NN denotes nearest neighbours, NNN-next-nearest neighbours and NNNN-next next nearest neighbours to the point **r**.

The order parameter time evolution, eqn (4), is numerically solved using a CDS^61,62^ for which the Laplacian is approximated as 
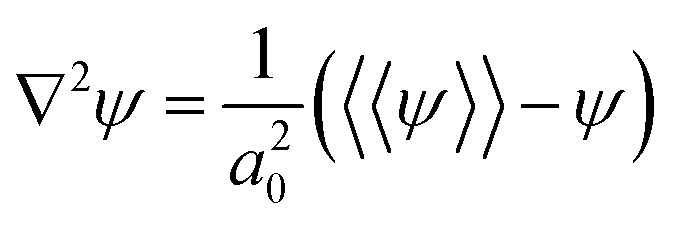
 where the term *a*_0_ represents the grid size unit and 〈〈*ψ*〉〉 is given by (ref. 61)6
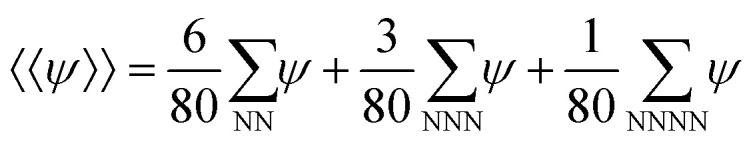
to calculate the isotropized Laplacian for a three-dimensional cubic system. NN, NNN, NNNN stand for nearest-neighbor, next-nearest-neighbor, and next-next-nearest neighbor, respectively, that is, summation over lattice points around the lattice point *ψ*_*ij*_ (see Fig. 3).

In order to compare simulations with experiments, it is necessary to use large simulation boxes, which can be reached thanks to the computational efficiency of the CDS model and a parallel implementation of the algorithm.^63,64^

### 2.1 Observables

The following quantities are calculated from the simulation data.

Structure factor *S*(**q**,*t*) = |*ψ*_**q**_(*t*)|^2^ is used to analyse the morphology of the BCP melt, with *ψ*_**q**_(*t*) being the Fourier transform of the order parameter *ψ*. In particular, the 2D structure factor can be calculated from the 2D slice at a distance *z* = *L*_*z*_/2 in the middle of the thin film. The corresponding 2D structure factor *S*(*q*_*xy*_,*ϕ*) can be averaged radially to obtain the angular scattering function *S*(*ϕ*) where *ϕ* is the polar angle in the *x*−*y* plane.

Mean absolute value of the order parameter 〈|*ψ*|〉 provides information on the degree of segregation of the BCP melt.^65^ A disordered, homogeneous BCP state is characterised by *ψ* = 0, while a phase-separated BCP, in the bulk, takes values |*ψ*| ∼ *ψ*_eq_. This global parameter 〈|*ψ*|〉 provides information on the degree of phase separation of the BCP over time.

Nematic order parameters *S*_*α*_ = 〈(3(**P̂**(**r**)**n̂**_*α*_)^2^ − 1)/2〉 describe the nematic order of the BCP with each axis *α* = *x*,*y*,*z* to provide information on the nematic alignment of the BCP with respect to the external shear flow, which is applied in the *x* direction. The local orientation of the lamella domains is calculated as **P̂**(**r**) = ∇*ψ*/(|∇*ψ*|) and the average is weighted with (∇*ψ*)^2^.

## 3 Results

**Fig. 4:**
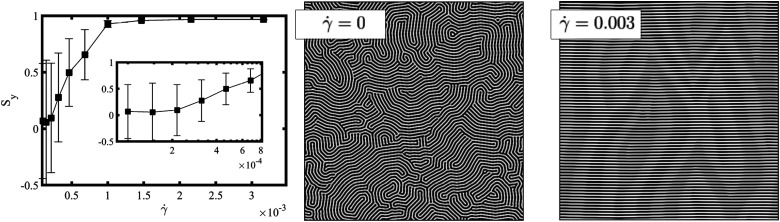
Left figure shows the nematic alignment *S*_*y*_ in the *y*-direction as a function of the applied shear rate for a film of *L*_*z*_/*H*_0_ = 1 with a thickness equal to the lamellar periodicity. Each point is averaged over 10 independent runs with different initial conditions. The inset figure shows the zoom of the nematic alignment for weaker shear flow rates. The figure in the centre shows the shear flow plane view when *γ̇* = 0, whilst the figure on the right shows the shear flow plane view when *γ̇* = 0.003. The system is initialised from a randomly oriented lamellar morphology with a high density of defects (see top-view – shear flow plane – a 2D snapshot for *γ̇* = 0), and shear is applied in the *x*-direction (see snapshot for *γ̇* = 0.003).

All simulations were performed using periodic boundary condition in the *x* and *y* direction with *L*_*x*_ = *L*_*y*_ = 512 grid points and a linear shear flow applied in the *z* direction, with *L*_*z*_ grid points for the film thickness. In the *z* direction we have applied neutral wall conditions *ψ*_S_ = 0 to describe no preference to the diblock copolymer blocks (see Fig. 2 for a schematics representation of the studied system.) The parameters used in the simulations are: *f* = 0.5, *u* = 0.5, *D* = 0.5, *B* = 0.02; these reproduce a symmetric lamellar system. The degree of segregation of the lamellae will be explored *via τ* which, for the parameters shown above, can be related to the Flory–Huggins parameter as^58^*χN* ≈ 3.6 + 34.6*τ*. The mobility is set to *M* = 1. Lengths and times are expressed in simulation units, *L* and *T* respectively. A grid size *a*_0_ = 1 and time discretisation δ*t* = 0.1 are used. In this work we investigate the mechanism that the lamellar domains undergo to align along the shear-flow direction. Fig. 4 shows the nematic alignment *S*_*y*_ along the *y*-direction upon the application of a shear flow with rate *γ̇*, for a system with *L*_*z*_/*H*_0_ = 1 and *τ* = 0.4. The system is initialised from a randomly equilibrated configuration (see 2D top-view snapshot for *γ̇* = 0), which results in a random nematic orientation for small shear rates *S*_*y*_ ∼ 0. The curve closely resembles the experimental behavior depicted in Fig. 2e of ref. 37. It demonstrates a distinctive transition from a randomly oriented lamella, consisting of multiple grains with random orientations and a high number of defects, towards a plateau where a single grain alignment along the direction of the shear stress dominates (perpendicular to the shear flow plane). For shear rates above a certain critical value *γ̇* ∼ 10^−3^ (corresponding to Wi ∼ 6.3 × 10^−4^), the lamellar domains align along the shear direction, as shown in the 2D snapshot in Fig. 4 for *γ̇* = 0.003. The perpendicular alignment of lamellar domains along the flow direction (and perpendicular to the shear plane), has been found in film experiments^37^ and in DPD simulations.^39^

**Fig. 5:**
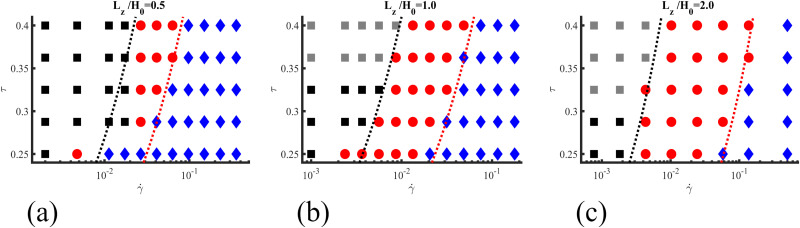
Diagram of the main mechanism of lamellar alignment with neutral walls under shear rate *γ̇* for a BCP with segregation given by *τ*. Three film thicknesses are considered, *L*_*z*_/*H*_0_ = 0.5, 1 and 2, respectively in (a), (b) and (c). The dotted lines represent curves of constant Weissenberg number Wi and can be seen to roughly correspond to the boundaries between different main mechanisms. Four main mechanisms can be distinguished: partial melting as blue diamonds, nucleation and growth as red circles, and rotation in 2D and 3D, respectively shown as black and gray squares.

Fig. 5 shows the different mechanism of alignment of the lamellar systems under shear flow in terms of the shear rate *γ̇* and the temperature-like parameter *τ*. The initial configuration of the lamellar morphology is similar to the method performed in ref. 33 and 56 where a pre-aligned lamellar system with two main grains oriented roughly perpendicular to the applied shear direction and to the shear flow plane has been applied. The presence of the ordered grains, grain boundaries and defects helps to initiate the different alignment mechanism. All systems have been equilibrated for 2 × 10^4^ steps, followed by the application of a shear flow in the *x* direction. The shear flow can align the lamellae system *via* three main mechanisms: rotations, nucleation and growth (NG) and selective disorder or partial melting (PM),^33,34,56^ denoted respectively by squares, circles and rhombi. Three different lamellar thicknesses have been simulated: *L*_*z*_/*H*_0_ = 0.5, 1, and 2, to investigate the role of vertical confinement (see Fig. 5). Dotted lines represent curves of constant Weissenberg number Wi and can be seen to roughly correspond to the boundaries between different main mechanisms, which are the primary factors in the alignment of the lamellae. While the main mechanisms dominate, other mechanisms are always present but with less prominence.

This suggests that the ratio of relaxation time scale of the BCP and the external shear flow determines the dominant mechanism of alignment.

The differences across the three diagrams in Fig. 5 reveals the role of the thin film thickness: a lamellar phase in a thin film with small thickness at *L*_*z*_/*H*_0_ = 0.5 is highly frustrated, which results in a quasi-2D behaviour for the alignment mechanism. A similar mechanism diagram has been found for BCP under electric fields.^28–30,33,34,55,56,66^ When the shear flow is weak only a rotation mechanism can be observed (black squares) in Fig. 5. For intermediate shear rates, nucleation and growth (red circles) is identified while for larger shear rates the system undergoes partial melting (blue diamonds).

When the thickness of the film is increased an extra mechanism (3D rotation) is found. The 3D rotation mechanism is indicated by grey squares in Fig. 5b and c. This new mechanism had not been observed previously, either in the 2D computational simulations or by experiments when an electric field was applied.^28–30,34,55,56^ This mechanism is only observed when the system is far above the order–disorder transition. Fig. 5 also shows that the region of the nucleation and grow mechanism is larger for thicker films (see Fig. 5c, red circles). In the rest of this section, we quantify each of the mechanisms and their differences.

**Fig. 6:**
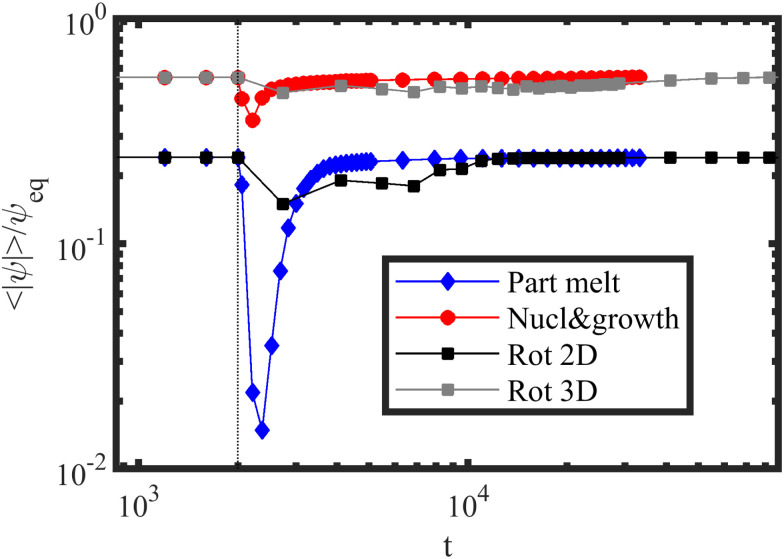
Time evolution of the mean absolute value of the order parameter 〈|*ψ*|〉/*ψ*_eq_ scaled with the equilibrium bulk value for four representative simulations corresponding to the phase diagram in Fig. 5(b) for *L*_*z*_/*H*_0_ = 1 with: *τ* = 0.25 and *γ̇* = 0.0316 (partial melting – PM); *τ* = 0.4 and *γ̇* = 0.0316 (nucleation and growth); *τ* = 0.25 and *γ̇* = 0.001 (rotation in 2D); and *τ* = 0.4 and *γ̇* = 0.001 (rotation in 3D). The vertical dotted line indicates the start point of shearing at *t* = 2000.

To better understand the different mechanisms a time evolution of the mean absolute value of the order parameter *ψ* is shown in Fig. 6, with 〈|*ψ*|〉 ≪ *ψ*_eq_. The vertical line in Fig. 6 shows the starting of the shear flow application. For rotation mechanisms, the order parameter is roughly constant, indicating little changes in the degree of separation during the mechanism. A considerable drop is shown in Fig. 6 for the blue curve (PM) indicating an increased disorder present in the partial melting while the other mechanisms display a better order during the time evolution of the system. The PM mechanism can also be identified using Minkowski functionals as shown in Fig. S1 in the ESI.†

**Fig. 7:**
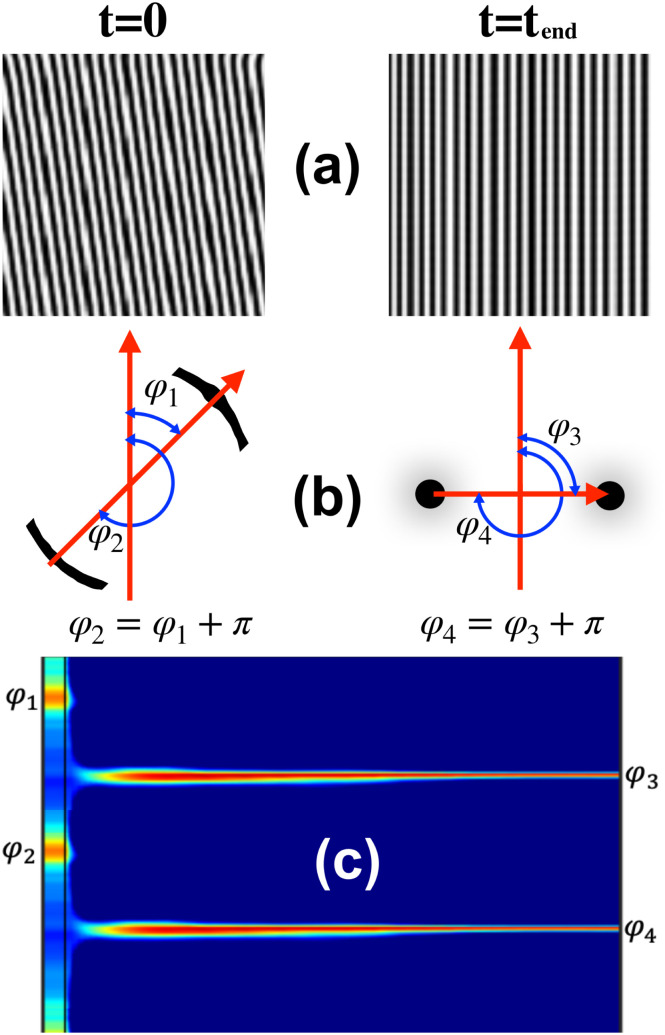
Shows a cartoon representation on the study of the different mechanisms using the azimuthal angles. The study starts from the real simulation time (a) for *t* = 0 with no shear and *t*_end_ after the simulation has concluded with an applied shear. In (b) the scattering peaks are represented with the relative angles and (c) represents the azimuthal angle of the main scattering peak over time. The different colours indicates the intensity of the scattering peaks.

The alignment mechanism has been examined through the azimuthal angle of the scattering peaks. A brief representation of how the azimuthal angle was calculated is shown in Fig. 7. The analysis begins with real-time simulations (see Fig. 7a), followed by an investigation of the scattering peaks (an example is shown in Fig. 7b). The angle and intensity of these peaks for each azimuthal angle over time are then reported in detail in Fig. 7c.

**Fig. 8:**
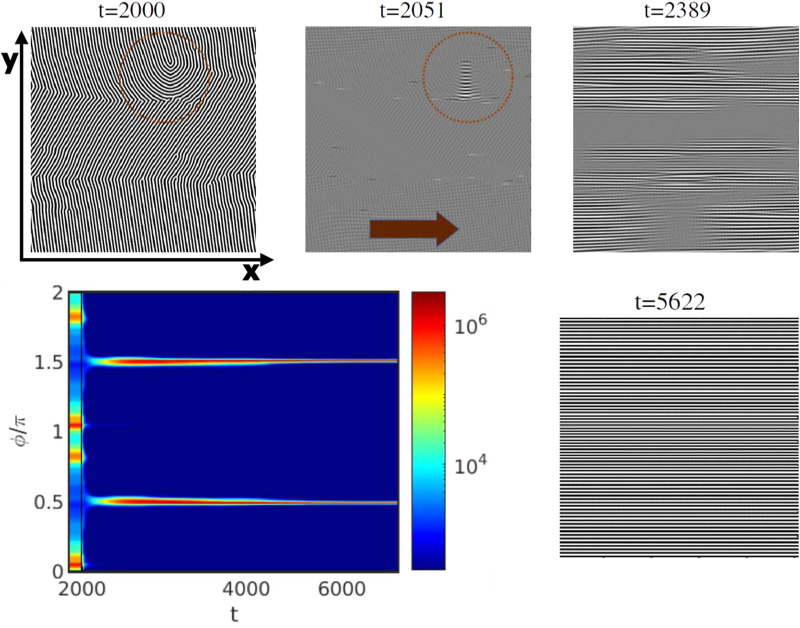
Alignment of a lamellar mesophase *via* PM with *τ* = 0.29, and *L*_*z*_/*H*_0_ = 1 for a shear rate *γ̇* = 0.18 (Wi = 1.1). The scattering intensity *S*(*ϕ*) is shown along with the representative snapshot of the *x*–*y* plane at *z* = *L*_*z*_/2. The red arrow indicates the direction of the shear flow. *ϕ* is the azimuthal angle of the main scattering peak and the time is shown in logarithmic scale. The colorbar indicates the intensity of the scattering peaks in logarithmic scale. The initial configuration, before applying the shear is shown for *t* = 2 × 10^3^.

The alignment pathway for a representative system undergoing the PM mechanism with *τ* = 0.19 and *γ̇* = 0.18 for a *L*_*z*_/*H*_0_ = 1 is shown in Fig. 8. The phase diagram of the PM mechanism has been shown in Fig. 5(b). Before the application of the shear flow (*t* = 2000), four scattering intensity peaks are visible *S*(*ϕ*), corresponding to the two grains of lamellar orientation. In all the scattering intensity plots, each peak exhibits a reflection at an angle of π, a feature that arises from the nematic nature of the lamella alignment (see Fig. 7b). This π symmetry causes each grain orientation in the lamellar mesophase to appear twice in the scattering intensity plots (see Fig. 7c). Upon the application of the shear flow in the direction of the red arrow in Fig. 8 (*t* = 2051), these four initial peaks fade, followed by a short period without any clear peak. This corresponds to the partial melting of the system, as also seen by the drop in 〈|*ψ*|〉 shown in Fig. 6. Rapidly, two new peaks re-emerge, shown in Fig. 8 and corresponding to the shear-aligned orientation of the lamellae.

**Fig. 9:**
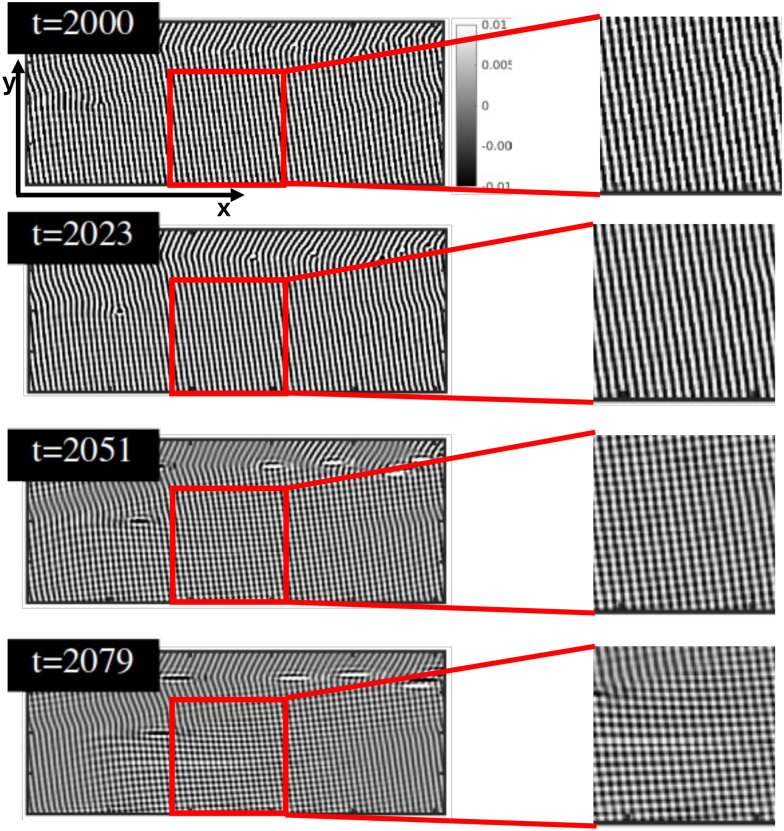
Chessboard-like transient configuration of the bottom part of the snapshots in Fig. 8. Snapshots show the bottom part of the full system, displaying the 2D slice at *z* = *L*_*z*_/2. Note that the limits of the colormap are smaller (*ψ* = 0.01) than for the main text corresponding image 8. The right side of the figure shows a zoom of the red square on the left.

2D snapshots of the top-view on the *x*–*y* plane at *z* = *L*_*z*_/2 in Fig. 8 indicate the formation of a homogeneous region (in gray), corresponding to melting. Only lamellae that were initially aligned with shear flow (grain boundaries and defects) remain not melted, indicated by the red circle in Fig. 8 (*t* = 2051). Fig. 9 shows a closer look at the order–disorder–order revealing the emerging of a transient morphology before alignment of lamellae perpendicular to the shear plane. In order to reveal the pattern of the BCP, the limits of the colorbar are decreased to 0.01 which allows to identify (and explicitly for the first time - to our knowledge) a chessboard-like configuration as an intermediate state between the initial state and the alignment along the shear direction. Fig. 9 shows also the time evolution of the chessboard pattern. At *t* = 2000 there are no chessboard patterns as the shear flow has not yet been applied. The formation of the chessboard starts to be visible after *t* = 2051, only after 51 time steps from the application of the shear flow. A zoomed-in view, on the right side of Fig. 9 has been provided to better visualize the pattern. Such pattern for the transition, from unstable to stable grains, was proposed by Schneider *et al*.^39^ using particle-based DPD simulations in Fig. 26.

**Fig. 10:**
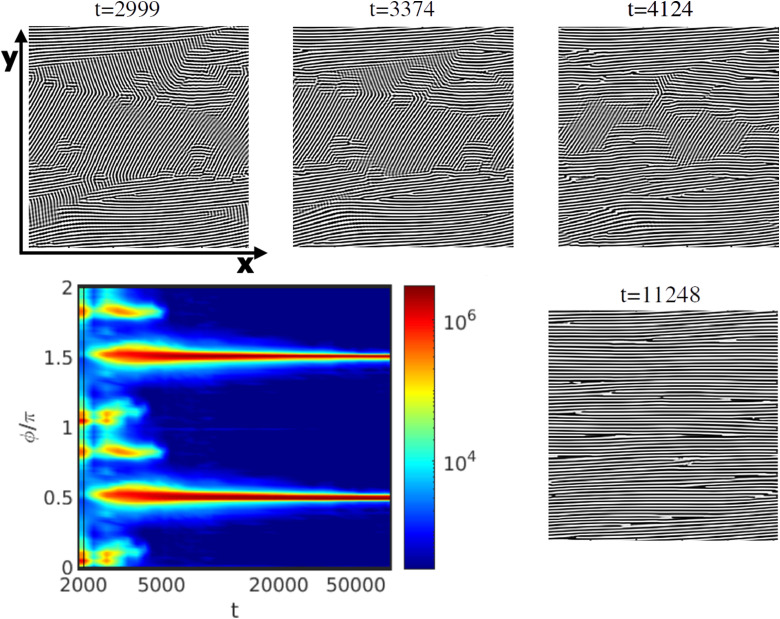
Alignment of a lamellar mesophase *via* NG with *τ* = 0.325, and *L*_*z*_/*H*_0_ = 1 for a shear rate *γ̇* = 0.013 (Wi = 6.2 × 10^−2^). The colourmap of the scattering intensity *S*(*ϕ*) is shown along with snapshots of the two-dimensional plane *x*–*y* at *z* = *L*_*z*_/2 (with time shown above each picture). The colorbar on the right indicates the strength of the scattering peaks, in logarithmic scale, with red areas indicating alignment peaks. The initial configuration is omitted, being the same as in Fig. 8.

An example of NG mechanism is shown in Fig. 10 under shear flow for *γ̇* = 0.013, *τ* = 0.325 and *L*_*z*_/*H*_0_ = 1. The shear started after a pre-aligned configuration obtained at *t* = 2000 (same as in Fig. 8). This can be seen from the scattering intensity peaks shown in Fig. 10. After the shear was applied, two main peaks are formed: one at the position of the old peak due to the pre-aligned system and a new one due the shear flow direction. While the simulation continues, the old peak loses intensity while the new peak grows in intensity (see scattering intensity between *t* = 2000 and 2300 in Fig. 10). After *t* = 2400 the system consists of lamellae domains oriented parallel to the shear flow direction and some domains with random orientation. It is still possible to identify some rotation of grains due to the change between the two peaks even though the main mechanism is given by the growth of domains of lamellae oriented parallel to the shear direction. As the nucleation and growth proceeds, the peaks which initially coexist together change till there are only domains left, parallel to the shear flow direction. Snapshots of Fig. 10 (see *t* = 3374 and 4124) show the decreasing of domains for lamellae non-parallel to the shear flow direction and after *t* = 11 000 only parallel domains to the shear flow directions remain. In this case, partial melting is not observed.

**Fig. 11:**
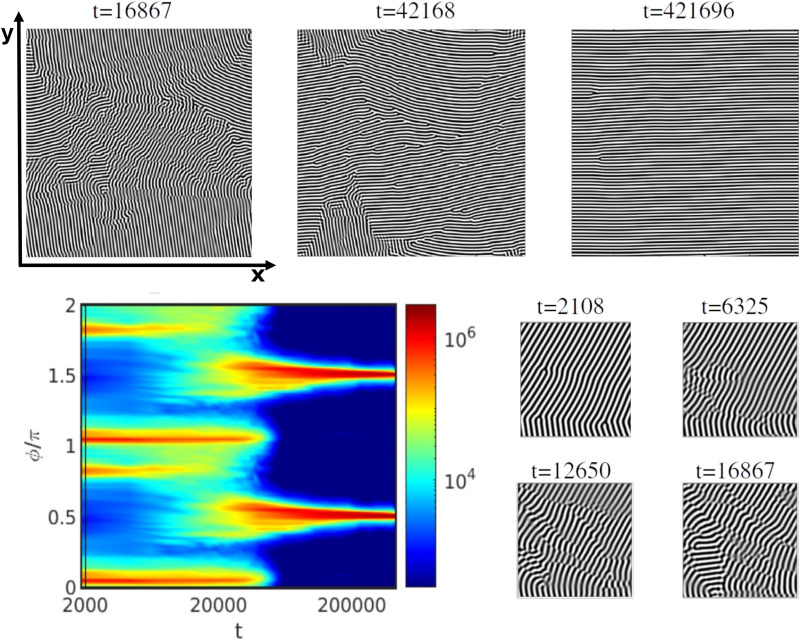
Rotation in 2D with *τ* = 0.29 and *L*_*z*_/*H*_0_ = 1 under a shear flow rate *γ̇* = 0.0024 (Wi = 1.4 × 10^−2^). The colourmap of the scattering intensity *S*(*ϕ*) is shown along with snapshots of the *x*–*y* plane *z* = *L*_*z*_/2 with time shown above each snapshot.

An example of the 2D rotation mechanism under an applied shear flow for *γ̇* = 0.0024 for *τ* = 0.29 and *L*_*z*_/*H*_0_ = 1 is shown in Fig. 11. The system is initialised from the same configuration as Fig. 8. This can be seen from the scattering intensity peaks shown in Fig. 11. Following the application of the shear flow, the two main peaks remain until *t* ∼ 10^4^ after which, they shift to *ϕ* = 0.5π (1.5π). The shifting of the intensity peaks corresponds to the rotation of grains. Fig. 11 inset shows an event of grain rotation starting from *t* = 2108 originated at defects in the lamellar morphology. Afterwards, defects move and merge to align parallel along the shear flow.

**Fig. 12:**
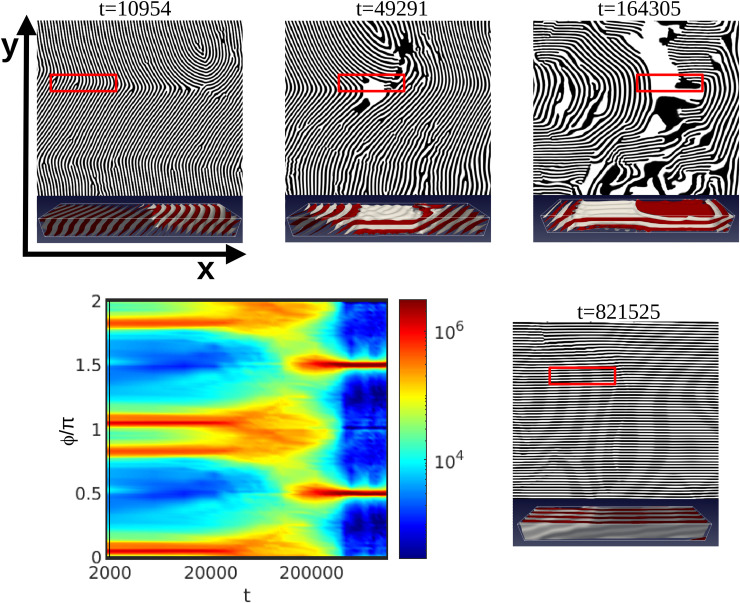
Rotation 3D for a system under a shear rate *γ̇* = 0.00183 and *τ* = 0.36 for a thicker film with *L*_*z*_/*H*_0_ = 2 (Wi = 7.1 × 10^−3^). The colourmap of the scattering intensity *S*(*ϕ*) is shown along with snapshots of the two-dimensional *x*–*y* plane at *z* = *L*_*z*_/2 with time shown above each snapshot. The respective top-view 3D images show a subset of the full system box.

Previous 2D studies^28,34,55,56^ have reported the same mechanism as the ones described so far: PM, NG and 2D rotation. However, the shear flow in a 3D system allows for non-2D mechanisms that involve vertical rotation. The new 3D rotation mechanism has been found for moderately thick films as shown in Fig. 5 (grey squares). Fig. 12 shows the scattering intensity for *γ̇* = 0.00183, *τ* = 0.36 for a *L*_*z*_/*H*_0_ = 2.0, which is similar to the mechanism shown in Fig. 11 for 2D rotation. The snapshots suggest the appearance of extended parallel lamellar domains (A or B polymer blocks described by black or white areas in Fig. 12). The time evolution of *S*(*ϕ*) of Fig. 12 shows that the main peaks do not disappear as in Fig. 8 and 10 or as peak shift as in Fig. 11. Before the shift of the peaks, in fact it is possible to notice the merging of the two main peaks while these are fading (around *t* = 200 000). However, the 2D scattering intensity is not sufficient to understand completely the out-of-plane alignment mechanism.

**Fig. 13:**
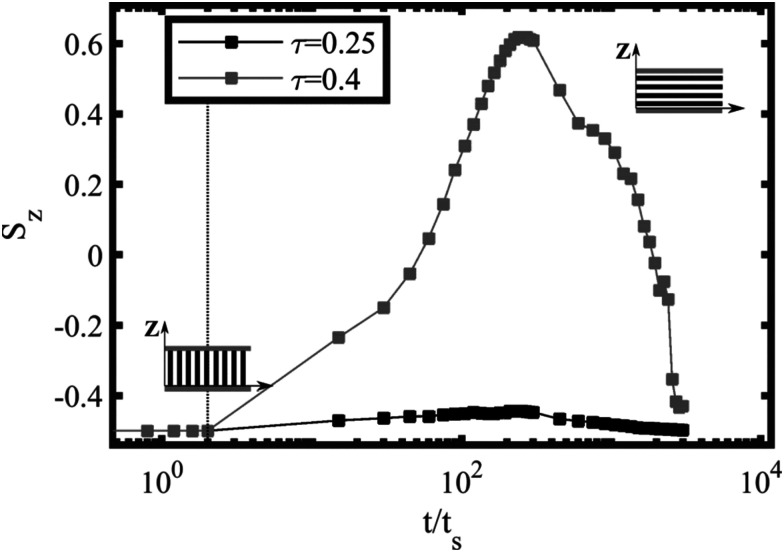
Nematic alignment *S*_*z*_ of the BCP perpendicular to the thin film for two representative runs with *L*_*z*_/*H*_0_= 2 and *γ̇* = 0.001 (see Fig. 5(c)): *τ* = 0.25 for the 2D rotation and *τ* = 0.4 for the 3D rotation mechanism. The early and long-time values of *S*_*z*_ ∼ −0.5 indicate perpendicular lamellar alignment (see miniatures) while *S*_*z*_ ∼ 0.6 indicate the partial transient parallel alignment of the lamellar domains. Vertical dotted line indicates the starting point of the shear flow, while the time is scaled with the characteristic shear time *t*_s_ = 1/*γ̇*.

Fig. 13 shows the vertical out-of-plane nematic ordering *S*_*z*_ for *γ̇* = 0.001 and two representative values of the temperature: *τ* = 0.25 and *τ* = 0.4, corresponding to 2D and 3D rotation. For the higher temperature, *τ* = 0.25 the system displays no significant change in the nematic order parameter, *S*_*z*_ ∼ −0.5, which indicates a prevailing perpendicular alignment of the lamellar domains throughout the film. For the lower temperature *τ* = 0.4, a clear change in sign in *S*_*z*_ ∼ 0.6 indicates that the lamellar domains are temporarily orienting parallel to the surfaces (along the shear plane). A 3D top-view of the out-of-plane rotation mechanism (shear plane) is shown in Fig. 12 for each snapshot, which was found experimentally and can be compared with AFM images in Fig. 4 of ref. 37 for films with comparable thicknesses.

## 4 Discussion

The reported results showed that the mechanisms of alignment of lamellar-forming BCP in thin films under shear are: PM, NG, and 2D and 3D rotation. The first three mechanisms have been previously found for BCP systems under electric fields.^29,30,33,34,55,56,66^ Moreover, the mechanism diagram shown in Fig. 5 is rather similar to the one illustrating lamellae under electric field as in Fig. 2 in ref. 56. Ignoring the distinction between 2D and 3D rotation, it is possible to determine the boundaries in the mechanism diagram based on the values of Wi: the dotted lines in Fig. 5 correspond to constant Wi values and roughly delimit the boundaries between different alignment mechanisms. A high value of Wi indicates that the relaxation time of the BCP is too large to accommodate the shear strain, and the system undergoes order–disorder–order transition. For smaller values of Wi, the shear stress can stabilise one of the domain orientations, while for very small Wi, the non-aligned grains can rotate. This roughly corresponds to the boundaries of the mechanism diagram in Fig. 5.

However for thin films with small thickness *L*_*z*_/*H*_0_ = 0.5, (Fig. 5(a)), the partial melting region (for low degree of segregation, small *τ*, bottom of the diagram) overlaps into the region of moderate Wi values. This can be due to surface effects, because of the proximity of the two neutral surfaces, which promote a lower segregation regime and therefore facilitates the order–disorder–order transition.

The 3D rotation shown in Fig. 5 is, to the best of our knowledge, a novel observation not previously documented in the literature. While lamellar alignment under electric fields has been extensively studied, it is important to note that alignment mechanisms under electric fields and shear flow are fundamentally different. Specifically, the electric field-induces alignment is reminiscent of extensional deformation under extensional flow condition. In contrast, the mechanisms observed under shear flow, such as the 3D rotation reported here, represent a distinct dynamic effect that cannot be directly related to alignment under electric fields. Experiments on films have shown lamellae aligned parallel to the surface under shear flow, as demonstrated in Fig. 4 of ref. 37. In our study, parallel lamellar alignment becomes prominent when the 3D rotation mechanism is present, particularly for thicker films (similar to experiments), as illustrated in Fig. 5(b) and (c). This alignment is more pronounced for thicker films with *L*_*z*_/*H*_0_ = 2 and occurs at higher values of *τ*. The parallel alignment of lamellar domains along the shear flow (and, in this context, parallel to the shear flow plane) has been identified as metastable when compared to the perpendicular alignment.^39^ Analytical studies have shown that for bulk systems, perpendicular and parallel lamellar orientations exhibit distinct stability regions depending on temperature and shear rate.^40^ However, for thin films under shear flow, analytical results are limited to systems with selective walls.^38^ The considerable similarity between the mechanism diagram for BCP under shear and under electric fields suggests that the alignment mechanism of lamellar-forming BCP under external fields are universal for thin films with small thickness, and intrinsic to the material. Our findings suggest that the observed 3D rotation (mechanism that can be explained by the specific 3D nature of the shear flow) introduces a novel mechanism that drives lamellar alignment, distinct from those induced by electric fields or previously-known effects in shear flow.

## 5 Conclusions

In conclusion, our study has revealed that the mechanisms governing the alignment of perpendicular lamellar-forming block copolymers (BCP) in thin films under shear flow are similar to those observed in BCP under electric fields. Moreover, we have found a new mechanism regarding the alignment of symmetrical lamellae on 3D films. Unlike 2D films under electric fields, 3D films provide an environment that facilitates a transient parallel alignment of the lamellar domains. This unique feature enables a novel mechanism called 3D rotation, which was previously not observed in 2D films under electric fields. Additionally, our study has shed light on the dynamics of the chessboard pattern, revealing a transition from unstable grain configurations to stable grains. This observation agrees with the proposed behaviour using particle-based DPD simulations, as shown in Fig. 26 in ref. 39. Our study has provided valuable insights into the alignment mechanisms of lamellar-forming block copolymers under shear, which can serve as a solid foundation for designing and optimising materials with desired properties. The observed 3D rotation mechanism in thicker films, the influence of surfaces on partial melting, and the dynamics of the chessboard pattern all represent exciting avenues for further exploration and validation through experimental studies.

## Author contributions

M. P. and J. D.: conceptualization, validation, data curation, formal analysis, project administration, investigation, methodology, writing – original draft, review & editing, software, supervision; J. D.: visualization; C. D.: validation, review; A. Z.: Supervision; I. P.: writing – review & editing, supervision.

## Data availability

Data for this article, including data to create Fig. 4–13 are available at [figshare] at https://doi.org/10.6084/m9.figshare.27276594.

## Conflicts of interest

There are no conflicts to declare.

## Supplementary Material

SM-021-D4SM01241K-s001
