# Supplementary material for: Mechanisms of alignment of lamellar-forming block copolymer under shear flow
Source: Soft Matter. 2024 Dec 16;21(3):476–87. doi: 10.1039/d4sm01241k (PMC11667677; doi:10.1039/d4sm01241k)
Supplement: SM-021-D4SM01241K-s001 [file SM-021-D4SM01241K-s001.pdf]

Supplementary Information (SI) for Soft Matter.
This journal is © The Royal Society of Chemistry 2024

Supplementary information

Marco Pinna, Javier Diaz, Christopher Denison, Andrei Zvelindovsky, and Ignacio Pagonabarraga

December 2024

1 Supplementary Information: Minkowski functionals

The order/disorder of the system can be visualised using the Minkowski functional (MFs) as shown in Fig. 1 and can be used to determine the details of the BCP phase transition. The MFs provide useful information on the BCP geometry and topological structure: the volume, surface, mean curvature, and Euler characteristic χ_E. The Euler characteristic χ_E takes distinct values depending on the topology of the BCP morphology for instance, $\chi_E = 1$ for a solid sphere, $\chi_E = 0$ for a torus, and negative χ_E values for a highly connected structure with holes (i.e., perforations, as in a perforated lamellar phase). Fig. 1 shows the 3 Minkowski functionals for the Euler characteristic(χ_E), Surface S and mean curvature. When the shear flow is above a threshold value we observe a drop in the Euler number and mean curvature while the surface is increasing. This behaviour describes the PM mechanism which is described in the MFs (see Fig. 1) by the diamond lines. A small χ_E number means that the morphology of the di-BCPs goes through a lot of new interconnected domains (perforations) creating new interfaces and decreasing the total curvature. The behaviour of the χ_E number decreases when the shear strength is increased while the surface area increases (see Fig. 1) respectively at time around W_i=10 and $W_i = 3$ for the χ_E and surface area. A similar χ_E number behaviour has been found in a previous study of BCPs under electric field using both CDS and the Dynamics Self Consistent Field Theory(DSCFT) computational techniques (see Fig. 4 in reference [1]). The rotation mechanisms represented by the squares (see Fig. 1) instead have higher χ_E numbers (at time around W_i=10) showing that there are less interconnected domains and hence the structure is more ordered during the time evolution.

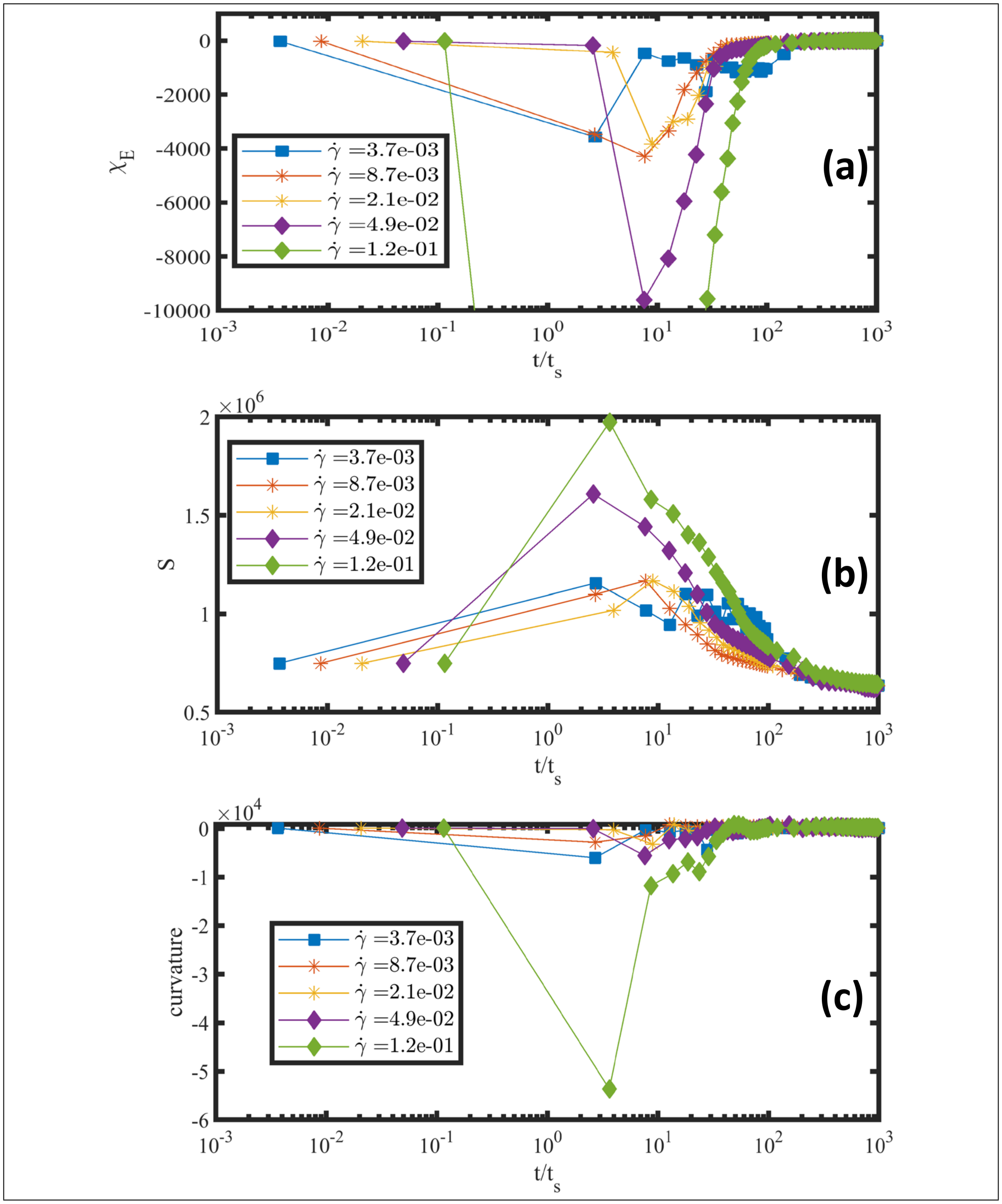

Figure 1: Time evolution of the Minkowski functionals for a lamella with $L_z/H_0 = 1.0$ and $\tau = 0.325$ for various values of $\dot{\gamma}$ spanning various mechanism of alignment. The three Minkowski Functional shown here are: (a) Euler characteristic, (b) surface and (c) mean curvature.

References

[1] GJA Sevink, M Pinna, KM Langner, and A V Zvelindovsky. Selective disordering of lamellae-forming diblock copolymers under an electric field. *Soft Matter*, 7:5161–5170, 2011.
